# 

**Published:** 1861-08

**Authors:** 


### CONTENTS.

ORIGINAL ESSAYS AND COMMUNICATIONS.

Inflammation. By W. H. ATKINSON,........................................ 437
Vulcanite Work,........................................................ 440
Influence of Association. By DR. J. G. HAMILL.......................... 442
The Amalgam Question,.................................................. 451
Concerning Tooth Extracting. By J. A. M'CLELLAND,...................... 457
Dr. J. Allen's Review of who are Dentists,............................. 463

SELECTIONS.

Dentition and its Derangements,........................................ 469
The American Dental Convention,........................................ 479
EDITORIAL.............................................................. 479

